# Glomerular filtration rate: new age- and gender- specific reference ranges and thresholds for living kidney donation

**DOI:** 10.1186/s12882-018-1126-8

**Published:** 2018-11-22

**Authors:** Anthony Fenton, Emma Montgomery, Peter Nightingale, A. Michael Peters, Neil Sheerin, A. Caroline Wroe, Graham W. Lipkin

**Affiliations:** 1 Department of Renal Medicine, University Hospitals Birmingham NHS Foundation Trust, Birmingham, UK; 2 Department of Renal Medicine, Newcastle upon Tyne Hospitals NHS Foundation Trust, Newcastle upon Tyne, UK; 3 Wolfson Computer Laboratory, University Hospitals Birmingham NHS Foundation Trust, Birmingham, UK; 4 Department of Nuclear Medicine, Brighton and Sussex University Hospitals NHS Trust, Brighton, UK; 5 Institute of Cellular Medicine, Newcastle University, Newcastle upon Tyne, UK; 6 Department of Renal Medicine, South Tees Hospitals NHS Foundation Trust, Middlesbrough, UK

**Keywords:** Glomerular filtration rate, Living kidney donation, Reference ranges

## Abstract

**Background:**

There is a need for a large, contemporary, multi-centre series of measured glomerular filtration rates (mGFR) from healthy individuals to determine age- and gender-specific reference ranges for GFR. We aimed to address this and to use the ranges to provide age- and gender-specific advisory GFR thresholds considered acceptable for living kidney donation.

**Methods:**

Individual-level data including pre-donation mGFR from 2974 prospective living kidney donors from 18 UK renal centres performed between 2003 and 2015 were amalgamated. Age- and gender-specific GFR reference ranges were determined by segmented multiple linear regression and presented as means ± two standard deviations.

**Results:**

Males had a higher GFR than females (92.0 vs 88.1 mL/min/1.73m^2^, *P* < 0.0001). Mean mGFR was 100 mL/min/1.73m^2^ until 35 years of age, following which there was a linear decline that was faster in females compared to males (7.7 vs 6.6 mL/min/1.73m^2^/decade, *P* = 0.013); 10.5% of individuals aged > 60 years had a GFR < 60 mL/min/1.73m^2^. The GFR ranges were used along with other published evidence to provide advisory age- and gender-specific GFR thresholds for living kidney donation.

**Conclusions:**

These data suggest that GFR declines after 35 years of age, and the decline is faster in females. A significant proportion of the healthy population over 60 years of age have a GFR < 60 mL/min/1.73m^2^ which may have implications for the definition of chronic kidney disease. Age and gender differences in normal GFR can be used to determine advisory GFR thresholds for living kidney donation.

**Electronic supplementary material:**

The online version of this article (10.1186/s12882-018-1126-8) contains supplementary material, which is available to authorized users.

## Background

The glomerular filtration rate (GFR) is a standard measure of renal function and can be determined by measuring the clearance of a molecule freely filtered through the glomerulus (measured GFR, mGFR). Although urinary inulin clearance is the gold standard method for determining mGFR, measuring the plasma clearance of ^51^Cr-EDTA is less cumbersome to perform and provides comparatively accurate results [1]. It is, therefore, the method used most commonly in the UK and throughout Europe, and is the technique recommended by the British Nuclear Medicine Society (BNMS) [2].

It is important to have accurate reference ranges for GFR. The current BNMS guideline quotes a reference range based on a series of mGFRs in 503 healthy subjects published in 1981: mean GFR in young adults was 105 mL/min/1.73 m^2^ which declined by 4 mL/min/1.73 m^2^ per decade up to 50 years of age, and 10 mL/min/1.73 m^2^ per decade thereafter [2, 3]. Similarly, in the largest study to date of mGFRs in a healthy population, which included 1057 prospective living kidney donors, mGFR declined by 4 mL/min/1.73 m^2^ per decade up to the age of 45 years, and 8 mL/min/1.73 m^2^ per decade thereafter [4]. A single-centre cohort of 904 prospective living kidney donors found the decline in mGFR with age was nonlinear, getting steeper with age [5]. Several studies have also reported gender to be a determinant of GFR [4, 6, 7]. It is important therefore that GFR reference ranges account for age and gender.

The purpose of this study was to use a large, contemporary, multi-centre series of mGFRs from healthy individuals to determine age- and gender-specific reference ranges. We also examined whether age and gender are determinants of GFR in a subgroup of individuals selected on the basis of having no evidence of kidney pathology. Further, we describe how our GFR reference ranges can be used to inform minimum thresholds of GFR considered safe for prospective living kidney donors to proceed to nephrectomy. This work formed the basis of new recommendations for the assessment of kidney function in the updated British Transplantation Society (BTS) guidelines on living donor kidney transplantation [8].

## Methods

This was a retrospective study using routinely-collected health data. The study population comprised prospective living kidney donors from UK renal centres who had undergone mGFR as part of their evaluation and were included irrespective of whether or not they proceeded to donation. Prospective donors with significant co-morbidity are excluded before the stage of having mGFR and therefore our study population can reasonably be regarded as representative of the healthy population.

We used hospital databases to identify prospective kidney donors who had undergone mGFR in three renal centres: Queen Elizabeth Hospital, Birmingham, 2007–2014; Freeman Hospital, Newcastle upon Tyne, 2009–2015; and James Cook University Hospital, Middlesbrough, 2005–2015. All prospective donors with mGFR during these years were included, and patient records were used to extract individual age, gender, and mGFR. These data from our three centres were then amalgamated with individual-level data from a published study of mGFRs in prospective living kidney donors from 15 other UK renal centres [9] (measured between 2003 and 2010, although the years of measurement varied between centres, and completeness of capture for each centre is not known), producing a dataset of prospective donors from 18 centres with the following variables: age, sex, renal centre, and mGFR per 1.73 m^2^ body surface area (BSA).

### GFR measurement

All 18 centres adhered to the BNMS guidelines for the measurement of GFR [2]. mGFR was determined by measuring the plasma clearance of a glomerular filtration tracer using the slope-intercept method with the Brochner-Mortensen correction applied [10]. The tracer used was ^51^Cr-EDTA in our three centres and in all of the other 15 centres apart from one which used ^99m^Tc-DTPA. The number of plasma samples obtained to calculate mGFR was two in our centres and varied between two to five in the 15 other centres. The Haycock formula, incorporating height and weight, was used to estimate BSA, and mGFR was scaled to 1.73m^2^ BSA [11].

### Statistical methods

Continuous variables are expressed as a mean and standard deviation. Reference ranges for GFR were created using segmented multiple linear regression incorporating the amalgamated data from all 18 centres to model mGFR as a function of age and gender. A normal probability plot was used to confirm the validity of the assumption that the residuals were from a Normal distribution. The upper and lower limits of the reference ranges were defined as two standard deviations (SD) above and below the mean, respectively. These ranges, in addition to other published evidence as described below, were used to establish GFR thresholds for prospective living kidney donors, below which kidney donation would not be recommended.

### Subgroup analysis

We identified a subgroup of prospective kidney donors from our three centres who had no proteinuria, normal renal imaging, and normal differential kidney function by collecting the following additional data from individual patient records: urine albumin-to-creatinine ratio (ACR) or protein-to-creatinine ratio (PCR), renal CT or MR imaging report, and differential kidney function by DMSA.

Prospective donors with any evidence of kidney pathology were excluded. Evidence of kidney pathology was defined as proteinuria (ACR ≥ 2.5 mg/mmol in males or ≥ 3.5 mg/mmol in females, or PCR ≥ 20 mg/mmol), abnormal renal imaging (other than angiomyolipoma, a few simple cysts, or a duplex system), or a > 10% difference in differential kidney function on DMSA. Those with missing data in any of these three variables were also excluded.

Data from the resultant subgroup of prospective living kidney donors with no evidence of kidney pathology were used to model mGFR as a function of age and gender as above.

## Results

### Study population and GFR reference ranges

**Fig. 1:**
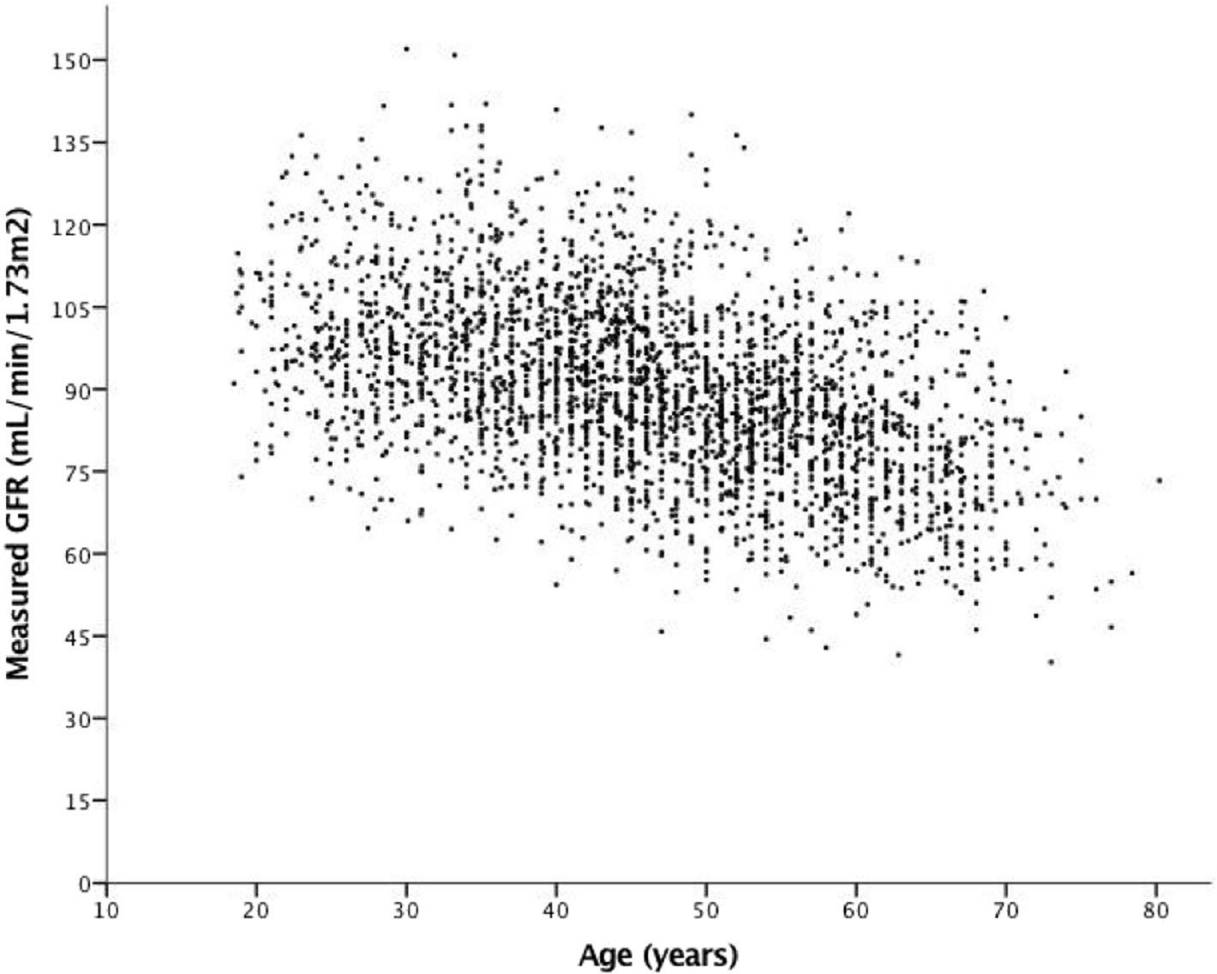
Scatter plot of measured GFR by age in 2974 prospective living kidney donors. GFR, glomerular filtration rate

We identified and collected data for 1096 prospective living kidney donors from our three centres who had undergone mGFR between 2007 and 2015. These data were amalgamated with individual-level data from 1878 prospective donors from the 15 other UK renal centres, producing a dataset for 2974 prospective donors from 18 centres. 1284 (43.2%) were male, mean age was 46.3 years (SD 12.3), and mean mGFR was 89.8 mL/min/1.73m^2^ (SD 15.8). The cohort included 459 (15.4%) individuals over 60 years of age, 51 (1.7%) individuals over 70 years, and 1 (0.0%) individual over the age of 80 years. Figure 1 shows a scatter plot of mGFR by age. Overall, 78 (2.6%) individuals had a mGFR < 60 mL/min/1.73m^2^. Forty-eight (10.5%) of the 459 individuals aged over 60 years had a mGFR < 60 mL/min/1.73m^2^.

**Fig. 2:**
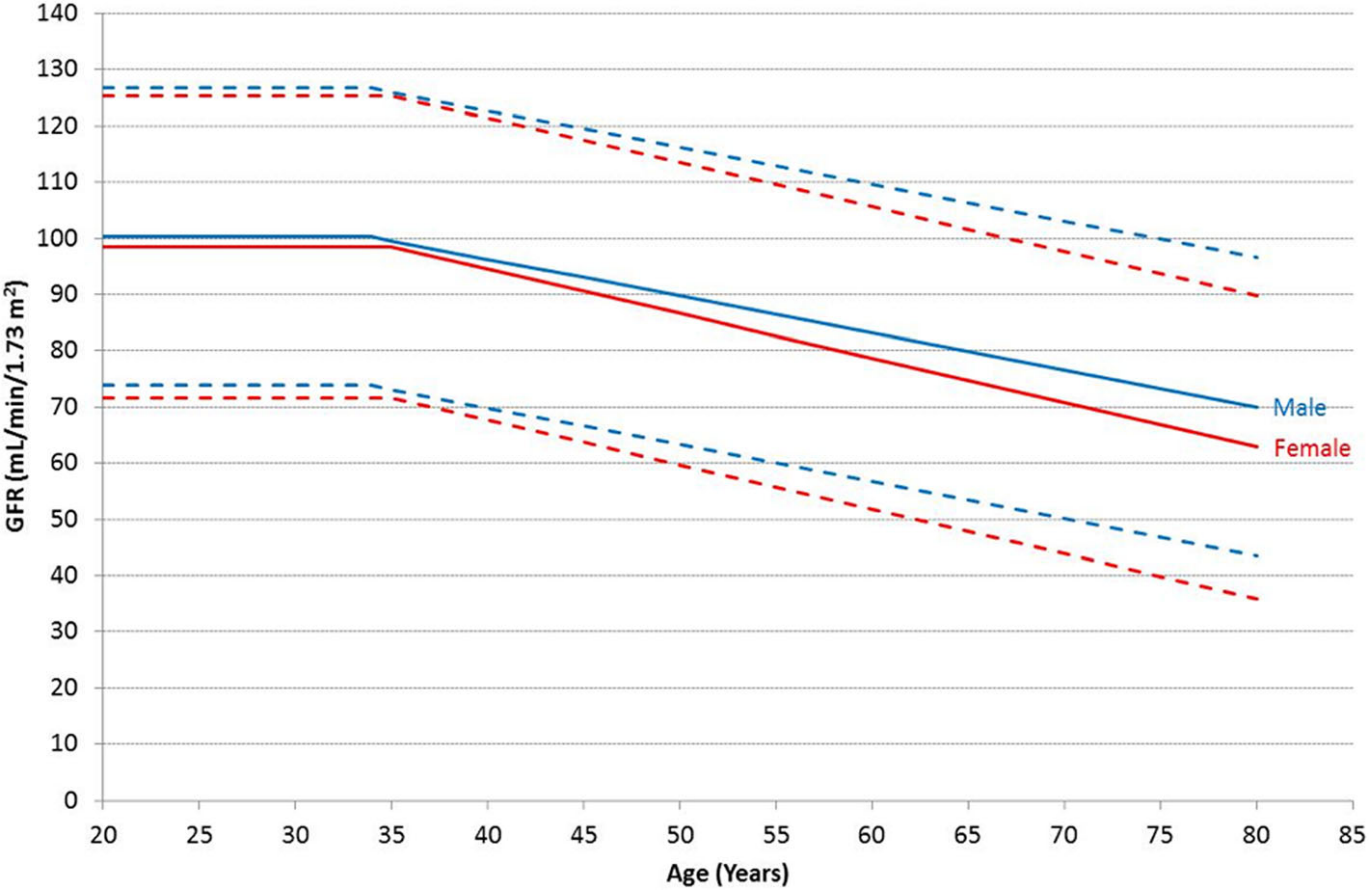
Age- and gender-specific GFR reference ranges based on measured GFRs from 2974 prospective living kidney donors. Solid lines represent mean GFR, and interrupted lines are two standard deviations above and below the mean. GFR, glomerular filtration rate

The means and reference ranges for GFR by age and gender are shown in Table 1 and Fig. 2. Overall males had a higher mGFR than females (92.0 vs 88.1 mL/min/1.73m^2^, *P* < 0.0001). Measured GFR was approximately 100 mL/min/1.73m^2^ until 35 years of age, following which there was a linear decline. This GFR decline was faster in females compared to males (7.7 vs 6.6 mL/min/1.73 m^2^/decade, *P* = 0.013). BSA did not correlate with age for males or females.

**Table 1:** Age- and gender-specific normal GFR reference ranges based on measured GFRs from 2974 prospective living kidney donors

Age^a^	Measured GFR^b^					
	Male			Female		
	Lower limit	Mean	Upper limit	Lower limit	Mean	Upper limit
20–34	74	100	127	72	99	125
35	73	100	126	72	99	125
40	70	96	123	68	95	121
45	67	93	119	64	91	117
50	63	90	116	60	87	114
55	60	86	113	56	83	110
60	57	83	110	52	79	106
65	53	80	106	48	75	102
70	50	77	103	44	71	98
75	47	73	100	40	67	94
80	43	70	97	36	63	90

*GFR* Glomerular filtration rate

^a^years

^b^mL/min/1.73m^2^

### Subgroup of prospective donors with no evidence of kidney pathology

Of the 1096 prospective donors in our three centres, 220 (20.1%) were excluded as they had missing data in one or more of the three following variables: ACR or PCR, renal imaging report, or DMSA result. Of the remaining 876 individuals with a complete dataset, 155 (17.7%) were excluded as they had evidence of renal pathology: 82 (9.4%) had a > 10% difference in differential kidney function on DMSA, 67 (7.6%) had abnormal renal imaging, and 25 (2.9%) had proteinuria (these total 174 because 19 of the 155 excluded individuals had two abnormal variables). Therefore, 721 prospective donors from our three centres were included in the subgroup analysis and their baseline characteristics are presented in Additional file 1: Table S1. Mean age was 45.7 (SD 12.5) years and 323 (44.8%) were male. Mean mGFR in the subgroup was significantly higher compared to those excluded from our three centres (91.6 ± 14.4 vs 85.6 ± 14.0 mL/min/1.73m^2^, *P* < 0.001). As in the total cohort of prospective donors, mGFR in the subgroup declined with age and the GFR decline after 35 years of age was more rapid in females. In addition, males had a higher initial mGFR which showed a continuous linear decline, unlike females who had a lower initial mGFR that was stable until 35 years of age before a linear decline. Mean GFR ± two SDs for the subgroup are shown in Additional file 1: Table S2 and Figure S1.

### Thresholds for living kidney donation

Age- and gender-specific mGFR thresholds, considered acceptable for prospective living kidney donors to proceed to nephrectomy, are shown in Table 2 and Additional file 1: Figure S2. We set a threshold mGFR of > 90 mL/min/1.73m^2^ for prospective donors younger than 30 years and a threshold of > 80 mL/min/1.73m^2^ for those aged 30–45 years. For those older than 45 years, the mGFR threshold was determined by calculating the lowest pre-donation mGFR that would leave the donor with a post-donation GFR within the reference range for their age and gender, assuming a 25% loss of GFR with donation. The rationale for these thresholds, which were determined based on our age- and gender-specific GFR reference ranges and previously published evidence, is discussed below.

**Table 2:** Advisory age- and gender-specific threshold GFRs considered acceptable for living kidney donation

Age^a^	Threshold GFR^b^	
	Male	Female
20–29	90	90
30–34	80	80
35	80	80
40	80	80
45	80	80
50	80	80
55	80	75
60	76	70
65	71	64
70	67	59
75	63	54
80	58	49

*GFR* Glomerular filtration rate

^a^years

^b^mL/min/1.73m^2^

## Discussion

This study is to the best of our knowledge the largest published series to date of mGFRs in a healthy population, and we have used it as the basis for new age- and gender-specific reference ranges for GFR and to define advisory mGFR thresholds for living kidney donation that form part of the updated BTS guidelines [8]. In 2974 prospective living kidney donors from 18 UK centres, we found that young adults had an mGFR of approximately 100 mL/min/1.73m^2^ until 35 years of age, following which there was a linear decline that was faster in females. An age-related decline in GFR, which was faster in females, was confirmed in a subgroup of prospective donors selected on the basis of no proteinuria, normal renal imaging, and normal differential kidney function.

### Ageing and GFR

Our finding that GFR is approximately 100 mL/min/1.73m^2^ in young adults is consistent with previously published data [3, 5, 12]. Published studies consistently show that GFR declines with age in healthy individuals, and it has been demonstrated that ageing is also associated with a decline in other physiological parameters, such as renal blood flow, and with structural changes such as a reduction in nephron number, glomerulosclerosis, and tubulointerstitial fibrosis [13–15].

Over 10% of prospective donors older than 60 years had a mGFR < 60 mL/min/1.73m^2^, and our model suggests that the lower limit of the normal range for GFR drops to 60 mL/min/1.73m^2^ by the age of 50 and 55 years for females and males, respectively, meeting the GFR cut-off in the current definition of chronic kidney disease (CKD) [16]. Our age- and gender-specific GFR reference ranges may be useful in the management of an older individual with a GFR < 60 mL/min/1.73m^2^: if GFR is within their reference range and there is no other evidence of kidney disease, one may be more confident in attributing a GFR < 60 mL/min/1.73m^2^ to normal ageing rather than disease, which may help to avoid unnecessary investigation and over-medicalisation. A meta-analysis which evaluated the interaction of age on the association between creatinine-based eGFR and end-stage renal disease (ESRD) and mortality suggested that a low eGFR is associated with increased risks across all age groups, although the relative mortality risk associated with a reduced eGFR decreased with increasing age [17].

It should also be noted that men younger than 55 years and women younger than 50 years could have a GFR below the lower limit of their reference range but still ≥60 mL/min/1.73m^2^ and so, in the absence of another marker of renal disease, would be missed by the current definition of CKD. The available data on long-term outcomes is insufficient to know whether a young individual in this category is at an increased lifetime risk of adverse health outcomes.

The definition and diagnosis of CKD, particularly in the older population, is an area of ongoing debate, and a discourse on the use of a single GFR cut-off is beyond the scope of this paper. However, several reviews and opinion pieces have suggested that greater emphasis should be placed on age-related changes in GFR than current definitions of CKD allow for [12, 18, 19]. Our data showing that over 10% of healthy adults over 60 years of age have a GFR < 60 mL/min/1.73m^2^ could be used to support that position.

### Gender and GFR

Although most studies have found no significant difference in GFR between males and females, our findings that males have a higher overall GFR and a less rapid decline with age are consistent with the results of several other reports [6, 20–22]. These findings were also evident in the subgroup of prospective donors with no evidence of kidney pathology.

Several theories have been proposed to explain the more rapid GFR decline seen in females. First, it has been proposed that females may have a higher GFR than males in young adulthood that is masked by scaling to BSA, which may lead to a faster rate of GFR decline similar to that seen in hyperfiltration-related renal pathology [9]. Second, as females age, the impact of oestrogens on renal haemodynamics and structure are lost due to a gradual decline in oestrogen levels even before the menopause [23–25]. There is no evidence that this faster rate of decline in females is detrimental to health, and in the UK the prevalence of ESRD in women is actually lower than in men at all ages [26].

### Minimum advisory GFR thresholds for living kidney donation

The evaluation of prospective living kidney donors aims to identify those whom donation would put at an unacceptably high risk of long-term complications, including ESRD. Previous studies have suggested that the risk of ESRD after living kidney donation is not higher than in the general population [27, 28], but there is a small absolute increased risk [29–31]. A recent meta-analysis found the relative risk for ESRD was about 9-fold higher in donors compared to non-donors, but the estimated incidence rate was less than 1 case per 1000 person-years [32]. Assessment of GFR in prospective kidney donors is an important factor in determining risk and living kidney donation guidelines have provided threshold GFRs above which the increased risk may generally be considered acceptable. For example, the 2017 KDIGO guideline suggests that a GFR ≥90 mL/min/1.73m^2^ is acceptable for donation, while a GFR < 60 mL/min/1.73m^2^ is not acceptable for donation and a GFR 60–89 mL/min/1.73m^2^ may be acceptable depending on other risk factors [33].

We recommended an advisory threshold GFR of > 80 mL/min/1.73 m^2^ for prospective donors aged 30–45, because this appears safe based on long-term outcome studies showing only a very small absolute increased risk of ESRD in cohorts of donors with this level of renal function and in this age range [30, 31]. However, because these studies contained only small numbers of younger donors, and another study showed an increased absolute lifetime risk in younger donors with a GFR < 90 mL/min/1.73m^2^ [34], we have recommended an advisory threshold GFR of > 90 mL/min/1.73m^2^ for donors younger than 30 years of age.

For those over 45 years, we have recommended advisory GFR thresholds based on calculating the lowest pre-donation GFR that would leave the donor with a post-donation GFR within our age- and gender-specific GFR normal ranges, assuming a 25% loss of GFR [27, 35, 36]. Based on data showing that age-related GFR decline after donation appears to be slower than in the general population, donors should remain within the healthy reference range up to the age of 80 years [27, 35, 37]. Whilst it is acknowledged that these may be considered arbitrary thresholds, from first principles it would seem sensible to aim to keep GFR in the normal range. Our thresholds, unlike those in the KDIGO guideline, would also allow some older donors with a GFR < 60 mL/min/1.73m^2^ to donate.

In our study population of 2974 prospective donors, these GFR thresholds would lead to the exclusion of an additional 5.0% (19.9% vs 14.8%) of prospective donors compared to the thresholds in the previous BTS living kidney donation guidelines if adhered to rigidly [38].

However, whilst threshold GFRs provide useful guidance to clinicians assessing prospective living kidney donors, individualised decision-making is important, especially in cases where GFR may be just below the recommended advisory threshold, or where there are compounding risk factors for ESRD. This has recently been facilitated by the development of an online tool (www.transplantmodels.com/esrdrisk) which provides a 15-year and lifetime pre-donation risk of ESRD in prospective donors, which was based on a meta-analysis of data from nearly 5 million healthy individuals, similar to kidney donor candidates, from general population cohorts [34].

The strengths of this study include the large size of the cohort and the fact that it incorporates individuals from multiple centres which increases diversity and the generalisability of our reference ranges. However, we recognise that our study has some limitations. First, it is likely that there is some variation in practice between the 18 centres in conducting mGFRs. There was variation in the number of blood samples taken to calculate mGFR, one centre used a different glomerular filtration tracer, and there may have been variation in pre-procedure advice given to individuals, such as that pertaining to diet, medications, and fasting.

Second, our estimates of GFR in the general population may be biased by the use of prospective living kidney donors as our study population. Individuals who have volunteered to donate a kidney and have had a satisfactory initial medical assessment to reach the point of having their GFR measured are likely to be healthier than the unscreened general population, and we would therefore anticipate a higher reference range than in the general population. Conversely, many prospective kidney donors are related to the intended recipient with renal failure, and therefore the proportion of individuals in our study population with a family history of renal disease is almost certainly higher than in the general population. This may have resulted in a lower estimate of GFR than truly exists in the general population. We did not have data on donor-recipient relationship to examine this further. Another consequence of using prospective kidney donors was that there were relatively few individuals in the cohort over 70 years of age, and this is to be borne in mind when interpreting our GFR reference ranges in this age group.

Third, our data are cross-sectional rather than longitudinal. The change in GFR that we describe with age is the change in mean GFR at population level and does not necessarily describe the expected change in an individual’s GFR with ageing. Indeed, previous work has shown that there is considerable variation in GFR decline with age [14, 39].

Finally, we did not have ethnicity data, which would have allowed us to validate previous work which has suggested that individuals of Asian ethnicity have a lower GFR [20, 40, 41]. Whether or not ethnicity is a determinant of GFR is an important question which requires further study in large, ethnically diverse cohorts, with accurate measures of GFR.

A large multi-centre observational cohort study with prospective recruitment of potential living kidney donors, incorporating baseline and longitudinal demographic and bioclinical data with a standardized method for mGFR and other renal phenotyping would be desirable to provide robust data on the effects of age, gender, and ethnicity on GFR.

## Conclusions

We have used a large multi-centre series of mGFR data from prospective living kidney donors to produce age- and gender-specific GFR reference ranges which have clinical utility during the assessment of potential living kidney donors and may have implications for the diagnosis of CKD in the general population.

## Additional file

Additional file 1: **Table S1.** Characteristics of subgroup of prospective living kidney donors from three centres with no proteinuria, normal renal imaging, and normal differential kidney function (*N* = 721). **Table S2.** Measured GFR (mean ± 2 SD) by age and gender in a subgroup of prospective living donors from three centres selected on basis of no proteinuria, normal renal imaging, and normal differential kidney function (*N* = 721). **Figure S1.** Measured GFR (mean ± 2 SD) by age and gender in a subgroup of prospective living donors from three centres with no proteinuria, normal renal imaging, and normal differential kidney function (*N* = 721). **Figure S2.** Advisory age- and gender-specific threshold GFRs for prospective living kidney donors to proceed to nephrectomy for males (A, blue) and females (B, red). (DOCX 258 kb)

## Abbreviations

ACR: Albumin-creatinine ratio
BNMS: British Nuclear Medicine Society
BSA: Body surface area
BTS: British Transplantation Society
CKD: Chronic kidney disease
CT: Computed tomography
DMSA: Dimercaptosuccinic acid
EDTA: Ethylenediaminetetraacetic acid
eGFR: Estimated glomerular filtration rate
ESRD: End stage renal disease
GFR: Glomerular filtration rate
KDIGO: Kidney Disease Improving Global Outcomes
mGFR: Measured glomerular filtration rate
MR: Magnetic resonance
PCR: Protein-creatinine ratio
SD: Standard deviation
UK: United Kingdom
