# Supplementary material for: Glomerular filtration rate: new age- and gender- specific reference ranges and thresholds for living kidney donation
Source: BMC Nephrol. 2018 Nov 22;19:336. doi: 10.1186/s12882-018-1126-8 (PMC6249883; doi:10.1186/s12882-018-1126-8)

**Supplementary Table 1.** Characteristics of subgroup of prospective living kidney donors from three centres with no proteinuria, normal renal imaging, and normal differential kidney function (*N*=721).

| **Characteristics** | | **Completeness**  **of data (%)** |
| --- | --- | --- |
| **Age (years)** | 45.7 (12.5) | 100 |
| **Gender (male)** | 323 (44.8) | 100 |
| **Ethnicity**  White  Asian  Black  Other | 451 (84.5)  68 (12.7)  10 (1.9)  5 (0.9) | 74.1 |
| **Measured GFR (mL/min/1.73m^2^)** | 91.6 (14.4) | 100 |
| **Estimated GFR (mL/min/1.73m^2^)***  MDRD  CKD-EPI | 87 (18)  94 (17) | 99.7 |
| **Body mass index (kg/m^2^)** | 26.6 (3.9) | 96.1 |
| **Differential kidney function**  Left (%)  Right (%) | 50.2 (2.5)  49.8 (2.5) | 100 |
| **Creatinine (µmol/L)** | 75 (15) | 99.7 |
| **Proteinuria (mg/mmol)**  ACR (*N*=516)  PCR (*N*=205) | <2.3 (<2.3, <2.3)  6 (4, 9) | 100 |
| **Anatomical variants**  **on renal imaging**  Cysts  AML  Duplex system | 82 (11.4)  10 (1.4)  3 (0.4) | 100 |
| **Hypertension** | 62 (9.2) | 93.8 |

Variables are presented as frequency (percent), mean (standard deviation), or median (interquartile range).

*Where ethnicity not known, no ethnicity correction applied, i.e. assumed to be non-black. ACR, albumin-creatinine ratio; AML, angiomyolipoma; CKD-EPI, Chronic Kidney Disease Epidemiology Collaboration; GFR, glomerular filtration rate; MDRD, Modification of Diet in Renal Disease; PCR, protein-creatinine ratio.

**Supplementary Table 2:** Measured GFR (mean ± 2 SD) by age and gender in a subgroup of prospective living donors from three centres selected on basis of no proteinuria, normal renal imaging, and normal differential kidney function (*N*=721).

| **Age^a^** | **Measured GFR^b^** | | | | | |
| --- | --- | --- | --- | --- | --- | --- |
|  | **Male** | | | **Female** | | |
|  | **-2 SD** | **Mean** | **+2 SD** | **-2 SD** | **Mean** | **+2 SD** |
| **20** | 84 | 108 | 132 | 74 | 99 | 123 |
| **25** | 81 | 105 | 129 | 74 | 99 | 123 |
| **30** | 79 | 102 | 126 | 74 | 99 | 123 |
| **35** | 76 | 99 | 123 | 74 | 99 | 123 |
| **40** | 73 | 96 | 120 | 71 | 95 | 120 |
| **45** | 70 | 94 | 117 | 67 | 92 | 116 |
| **50** | 67 | 91 | 114 | 64 | 88 | 113 |
| **55** | 64 | 88 | 111 | 60 | 85 | 109 |
| **60** | 61 | 85 | 109 | 57 | 81 | 106 |
| **65** | 58 | 82 | 106 | 53 | 78 | 102 |
| **70** | 55 | 79 | 103 | 49 | 74 | 99 |
| **75** | 52 | 76 | 100 | 46 | 71 | 95 |
| **80** | 50 | 73 | 97 | 42 | 67 | 92 |

^a^years; ^b^mL/min/1.73m^2^. GFR, glomerular filtration rate; SD, standard deviation.

**Supplementary Figure 1.** Measured GFR (mean ± 2 SD) by age and gender in a subgroup of prospective living donors from three centres with no proteinuria, normal renal imaging, and normal differential kidney function (*N*=721).

Mean GFRs represented by solid lines, and ± 2 SD represented as interrupted lines.

GFR, glomerular filtration rate; SD, standard deviation.

**Supplementary Figure 2.** Advisory age- and gender-specific threshold GFRs for prospective living kidney donors to proceed to nephrectomy for males (A, blue) and females (B, red).

A)


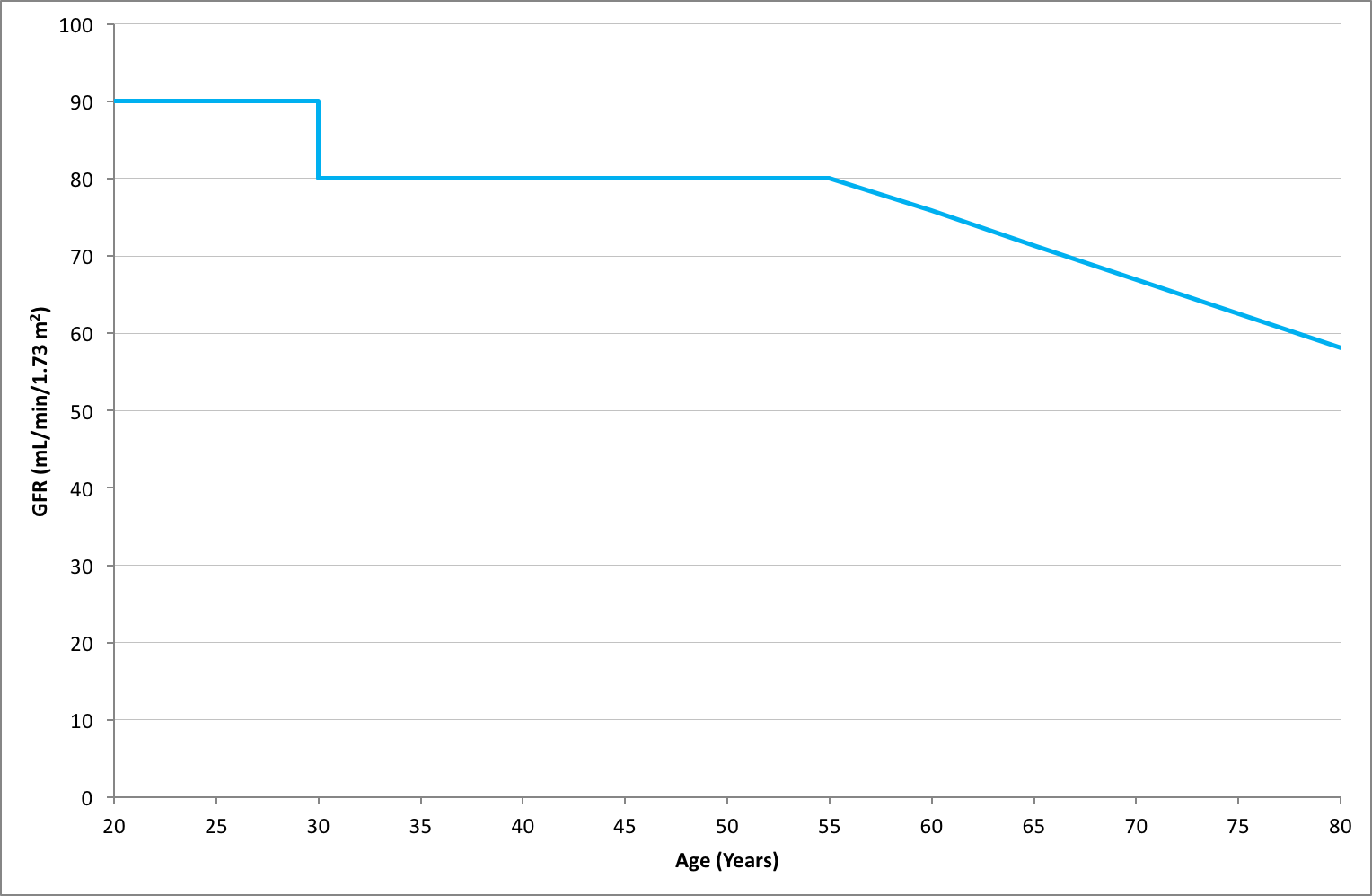


B)


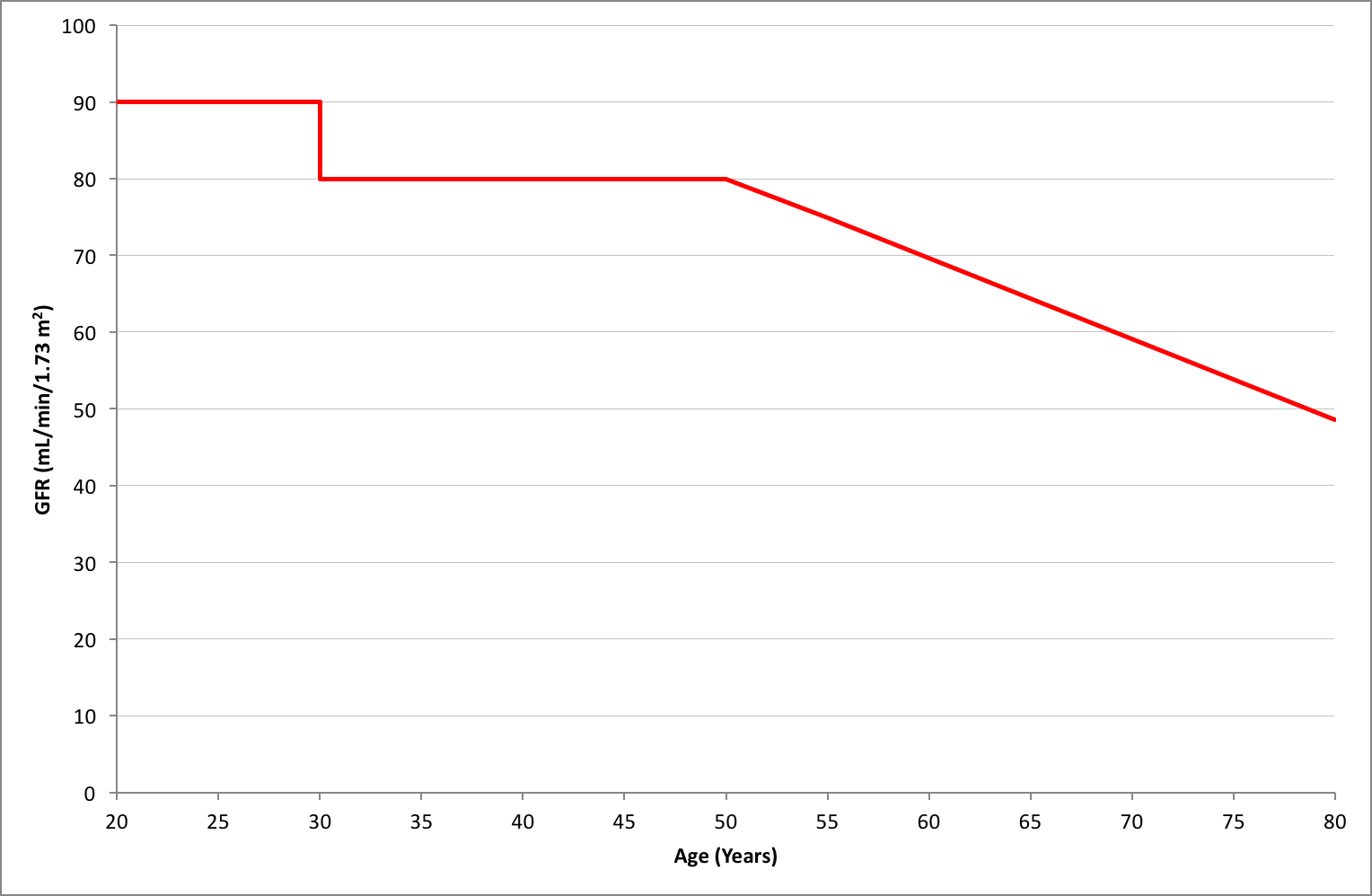

Supplement: Supplementary file 1 — Table S1. Characteristics of subgroup of prospective living kidney donors from three centres with no proteinuria, normal renal imaging, and normal differential kidney function (N = 721). Table S2. Measured GFR (mean ± 2 SD) by age and gender in a subgroup of prospective living donors from three centres selected on basis of no proteinuria, normal renal imaging, and normal differential kidney function (N = 721). Figure S1. Measured GFR (mean ± 2 SD) by age and gender in a subgroup of prospective living donors from three centres with no proteinuria, normal renal imaging, and normal differential kidney function (N = 721). Figure S2. Advisory age- and gender-specific threshold GFRs for prospective living kidney donors to proceed to nephrectomy for males (A, blue) and females (B, red). (DOCX 258 kb) [file 12882_2018_1126_MOESM1_ESM.docx]
